# Plant Resistance Inducers Suppress Anthracnose on Chili Fruit and Are Associated with Inducer-Specific Transcriptional Responses

**DOI:** 10.3390/plants15213257

**Published:** 2026-10-22

**Authors:** Pisut Keawmanee, Chutchamas Kanchana-udomkan, Yuwadee Sirirat, Santiti Bincader, Ratiya Pongpisutta, Natnicha Aonsri, Chainarong Rattanakreetakul

**Affiliations:** 1 Department of Plant Pathology, Faculty of Agriculture at Kamphaeng Saen, Kasetsart University, Nakhon Pathom 73140, Thailand; pisut.keaw@ku.th (P.K.); yuwadee.s@ku.th (Y.S.); natnicha.aon@ku.th (N.A.); 2 Tropical Vegetable Research Center, Department of Horticulture, Faculty of Agriculture at Kamphaeng Saen, Kasetsart University, Nakhon Pathom 73140, Thailand; chutchamas.k@ku.th; 3 Program in Plant Science, Faculty of Agricultural Technology and Agro-Industry, Rajamangala University of Technology Suvarnabhumi, Phra Nakhon Si Ayutthaya 13000, Thailand; santiti.b@rmutsb.ac.th; 4 Department of Plant Pathology, Faculty of Agriculture, Kasetsart University, Bangkok 10900, Thailand; ratiya.p@ku.th

**Keywords:** anthracnose, *Capsicum*, *Colletotrichum*, induced resistance, plant resistance inducers, RNA-seq

## Abstract

Anthracnose caused by *Colletotrichum* spp. is a major constraint to chili (*Capsicum* spp.) production in Thailand. We characterized isolates from nine provinces and evaluated five plant resistance inducers (PRIs). Seventy-four isolates were sorted into three morphological groups, and multilocus analysis (ITS, *ACT*, *CHS-1*) placed seven representative isolates in the *C. gloeosporioides* and *C. truncatum* species complexes. *C. truncatum* KRI015 and *C. gloeosporioides* RBR009 were the most aggressive isolates. On the fruit of six genotypes, salicylic acid (SA) and acibenzolar-S-methyl (ASM) significantly reduced anthracnose at 21 days post-inoculation in all five genotype × isolate combinations in which inducers significantly reduced disease (by 27–100%), whereas β-aminobutyric acid (BABA), chitosan (CHI) and *Bacillus subtilis* B01 were genotype- and application-dependent; the highly susceptible ‘Amphawa Gold’ was only partly protected (27–50%). ASM and foliar B01 increased soluble phenolics at all sampling times. RNA sequencing 24 h after treatment suggested inducer-specific responses: ASM up-regulated putative capsidiol-biosynthesis genes (six 5-epi-aristolochene synthase genes and *CYP71D7*), WRKY transcription factors and plant–pathogen interaction genes; CHI induced lignin- and cuticle-related genes and protease inhibitors; B01 repressed salicylate carboxymethyltransferase; and BABA-repressed defense genes, compatible with priming. SA and ASM are the most promising candidates for integrated anthracnose management in chili.

## 1. Introduction

Chili (*Capsicum* spp.) is one of the most economically important vegetable and spice crops in Thailand and worldwide. The leading producers of fresh chili include China, Indonesia, Mexico and Nigeria [1]. In Thailand, chili is grown on approximately 21,415 ha with an annual yield of about 251,655 t, and the country ranks third globally in dried chili exports [2]. Commercial cultivars are grouped by fruit size into large-fruited types (e.g., cayenne, banana and yellow peppers) and small-fruited bird’s eye chili, both of which have substantial market value.

Among the biotic constraints on chili production, anthracnose caused by *Colletotrichum* spp. is considered the most destructive disease in tropical and subtropical regions [3], and the genus ranks among the ten most economically important groups of fungal plant pathogens [4]. In Thailand, three species are predominantly associated with chili anthracnose: *C. truncatum*, *C. scovillei* (*C. acutatum* species complex) and *C. siamense* (*C. gloeosporioides* species complex) [3,5,6]. These hemibiotrophic pathogens cause sunken, water-soaked lesions on fruit at various developmental stages, with losses of marketable yield that can reach 50–80% [7]. Breeding for resistance is hampered by the complexity of host–pathogen interactions, the lack of commercially acceptable resistant *C. annuum* cultivars and the genetic plasticity of *Colletotrichum* populations [8]; climate change may further favor the emergence of more aggressive pathotypes.

Chemical fungicides remain the main management tactic [9,10]. Because chili fruit are often eaten raw, fungicide residues are a concern for consumer health, and repeated applications can select for fungicide-resistant *Colletotrichum* strains [9]. These challenges have increased interest in plant resistance inducers (PRIs) as low-residue alternatives [11,12].

Plants possess a multilayered immune system comprising constitutive barriers and inducible defenses that are activated when pattern recognition receptors perceive pathogen- or damage-associated molecular patterns [13,14]. Induced resistance is broadly classified into systemic acquired resistance (SAR) and induced systemic resistance (ISR). SAR is mediated by salicylic acid (SA) signaling, involving SA accumulation, nuclear translocation of the regulator NPR1 and up-regulation of pathogenesis-related (PR) genes such as *PR-1*, *PR-2* (β-1,3-glucanase) and *PR-3* (chitinase) [15,16]. ISR is triggered mainly by beneficial rhizosphere microorganisms and is mediated by jasmonic acid (JA) and ethylene (ET) signaling [15,17]. Simultaneous activation of SAR and ISR can have an additive protective effect [18], suggesting value in combining PRIs with different modes of action.

A central concept linking these pathways is defense priming, a physiological state in which the immune system responds faster and more strongly to subsequent attack without the fitness costs of constitutive defense activation [19,20,21]. Several chemical and biological PRIs act as priming agents or direct elicitors. SA is the primary endogenous SAR signal; acibenzolar-S-methyl (ASM) is a functional SA analog that activates SAR downstream of SA accumulation [22]; β-aminobutyric acid (BABA) is a non-protein amino acid that primes both SA-dependent and SA-independent defenses [23,24]; and chitosan (CHI), a deacetylated chitin derivative, acts as a PAMP-like elicitor that up-regulates defense genes including *NPR1*, *PR-1*, *LOX*, *PAL* and ERF4 [25,26,27]; in pepper, foliar SA and chitosan also trigger oxidative and defense-gene responses [28]. *Bacillus subtilis* and related species elicit ISR, produce antifungal lipopeptides (iturin, surfactin, fengycin) and have reduced *Colletotrichum* anthracnose in several crops [29,30,31].

Although *Colletotrichum* diversity in Thai chili has been partly documented [3,5], updated surveys across major production areas are limited. Moreover, abiotic and biotic PRIs have rarely been compared side by side in Thai chili germplasm [32], and the molecular basis of their effects in chili remains poorly understood. This study therefore aimed to (1) survey and identify the *Colletotrichum* spp. causing anthracnose in major chili-producing areas of Thailand; (2) evaluate SA, ASM, BABA, CHI and *B. subtilis* B01 for the suppression of anthracnose on chili fruit; and (3) characterize the host responses elicited by each inducer using RNA sequencing and total phenolic content analysis.

## 2. Results

### 2.1. Survey, Isolation and Morphological Classification of Colletotrichum spp.

Diseased fruit were collected from 14 sites in nine provinces between October 2024 and March 2025, and a total of 74 *Colletotrichum* isolates were recovered, most from Kanchanaburi (*n* = 24), followed by Nakhon Pathom (*n* = 10), Nakhon Ratchasima (*n* = 9) and Ratchaburi (*n* = 9) (Table 1). Bird’s eye chili was the most frequently sampled type, reflecting its dominance in commercial production.

**Figure 1:**
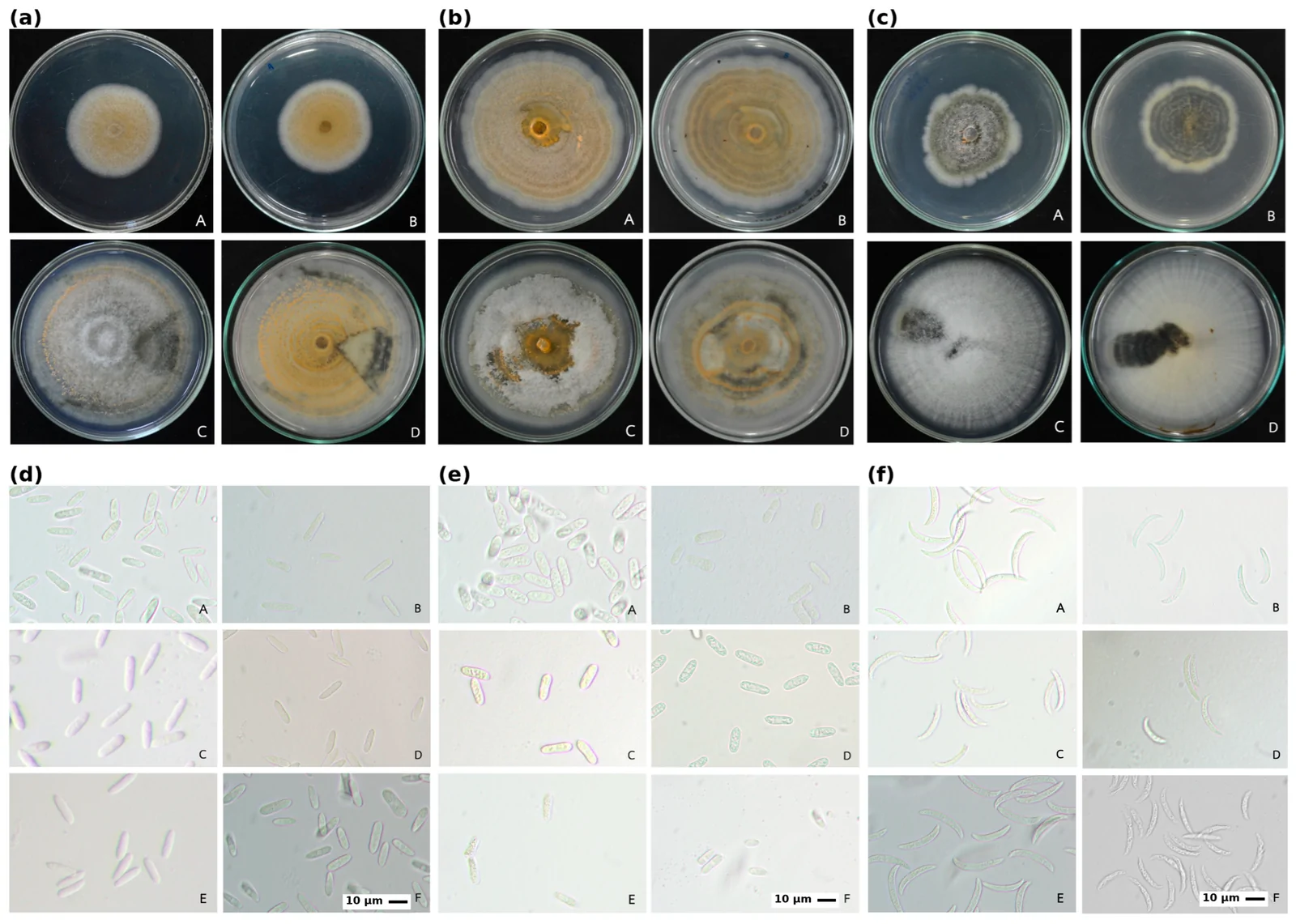
Colony and conidial morphology of representative *Colletotrichum* isolates on PDA after 7 days. (**a**) Group I, (**b**) Group II and (**c**) Group III colonies (four plates per panel); (**d**–**f**) conidia of Groups I–III, respectively. Plates in (**a**–**c**), front and reverse sides: (**a**) A, B, TAK004; C, D, PKN002; (**b**) A, B, KRI002; C, D, RBR009; (**c**) A, B, KRI012; C, D, KRI015. Conidia: (**d**) A, TAK004; B, NMA009; C, PKN002; D, PNB001; E, RBR008; F, KRI024; (**e**) A, KRI002; B, KRI017; C, RBR002; D, RBR009; E, NMA003; F, PKN004; (**f**) A, KRI012; B, KRI015; C, KRI023; D, RBR001; E, SKN002; F, PKN005. Scale bars in panel F of (**d**–**f**) = 10 µm.

After 7 days on potato dextrose agar (PDA), the isolates were sorted into three morphological groups on the basis of colony and conidial characters (Table 2; Figure 1). Group I (12 isolates; 16.2%) formed pale salmon-pink to orange colonies with fusiform conidia with acute ends (12–18 × 3–6 µm). Group II (33 isolates; 44.6%) formed gray-white to grayish-green colonies with orange conidial masses and cylindrical to ellipsoidal conidia (12–20 × 4–6 µm). Group III (29 isolates; 39.2%) formed gray to dark, irregularly pigmented colonies with falcate conidia bearing a truncate base (18–27 × 2.5–6 µm). These characters resemble those described for the *C. acutatum*, *C. gloeosporioides* and *C. truncatum* species complexes, respectively [33,34,35]; however, because morphology alone cannot reliably assign isolates to species complexes, complex membership was determined only by the multilocus analysis of representative isolates (Section 2.2).

### 2.2. Multilocus Phylogeny of Representative Isolates

**Figure 2:**
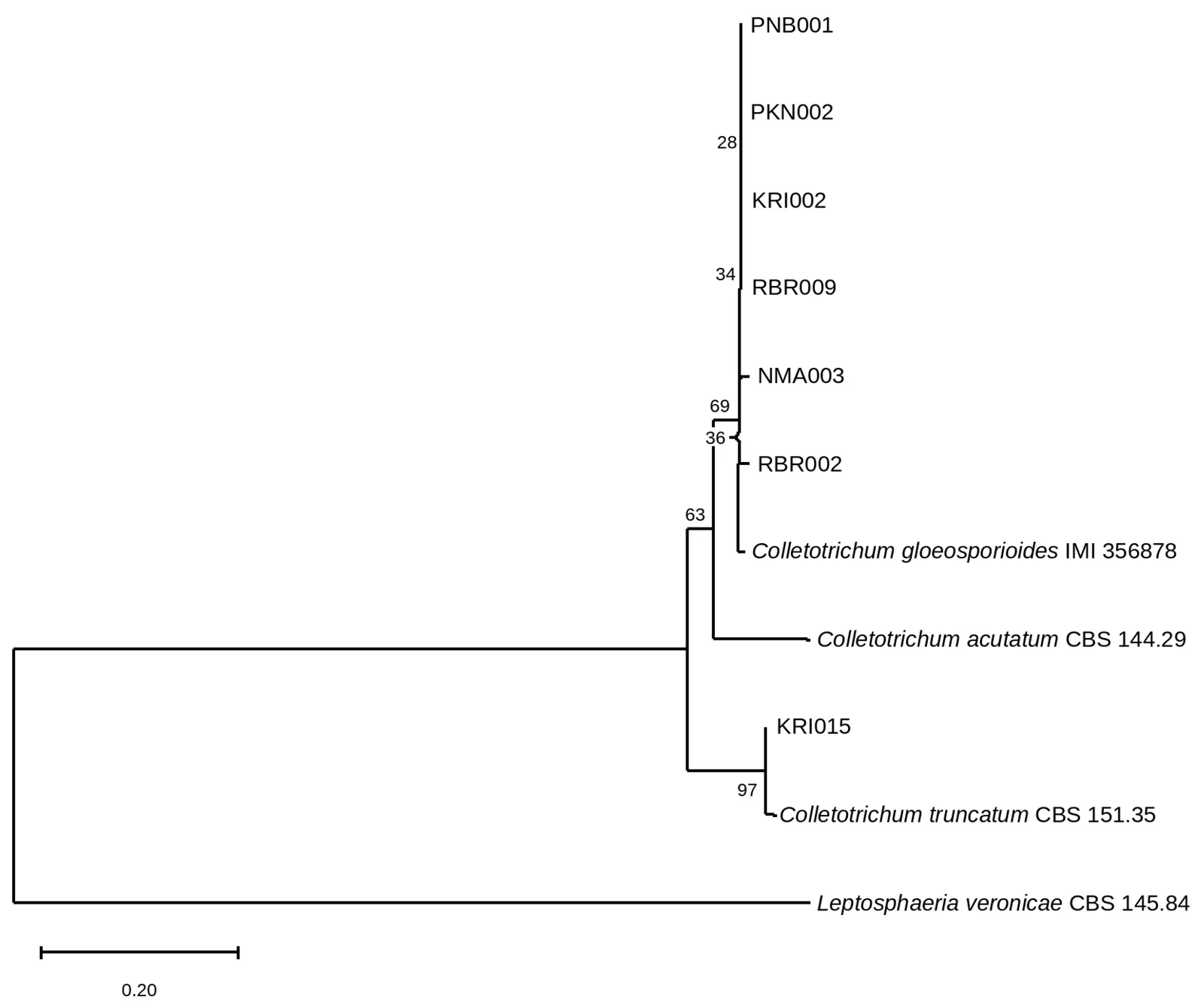
Neighbor-joining tree of seven *Colletotrichum* isolates from chili and reference strains based on concatenated ITS, *ACT* and *CHS-1* sequences. Bootstrap values (1000 replicates) are shown at the nodes. *Leptosphaeria veronicae* CBS 145.84 was used as the outgroup. Scale bar = substitutions per site.

Seven isolates were sequenced to represent the three morphological groups, five provinces and the range of virulence in the pathogenicity test, including the two most aggressive isolates (KRI015 and RBR009). Neighbor-joining analysis of ITS, actin (*ACT*) and chitin synthase 1 (*CHS-1*) sequences from seven representative isolates resolved two principal lineages (Figure 2). Isolate KRI015 clustered with *C. truncatum* CBS 151.35 in every single-locus tree (bootstrap support: ITS 92%, *ACT* 98%, *CHS-1* 95%) and in the concatenated tree (97%), identifying it as a member of the *C. truncatum* species complex. The remaining six isolates (KRI002, NMA003, PKN002, PNB001, RBR002 and RBR009) grouped with *C. gloeosporioides* IMI 356878 in all analyses, although support within this clade was low to moderate (28–82% in single-locus trees; 28–69% in the concatenated tree). Isolates PKN002 and PNB001, which belonged to morphological Group I (fusiform conidia), clustered with the *C. gloeosporioides* species complex, whereas the *C. acutatum* reference strain CBS 144.29 formed a separate clade (63%) (Figure 2). Thus, six of the seven sequenced isolates clustered with the *C. gloeosporioides* species complex and one with the *C. truncatum* species complex. Because only seven isolates were sequenced, the relative abundance of the species complexes in the whole collection could not be determined, and the percentages in Table 2 refer to morphological groups only.

### 2.3. Pathogenicity and Virulence of Isolates on Detached Fruit

**Figure 3:**
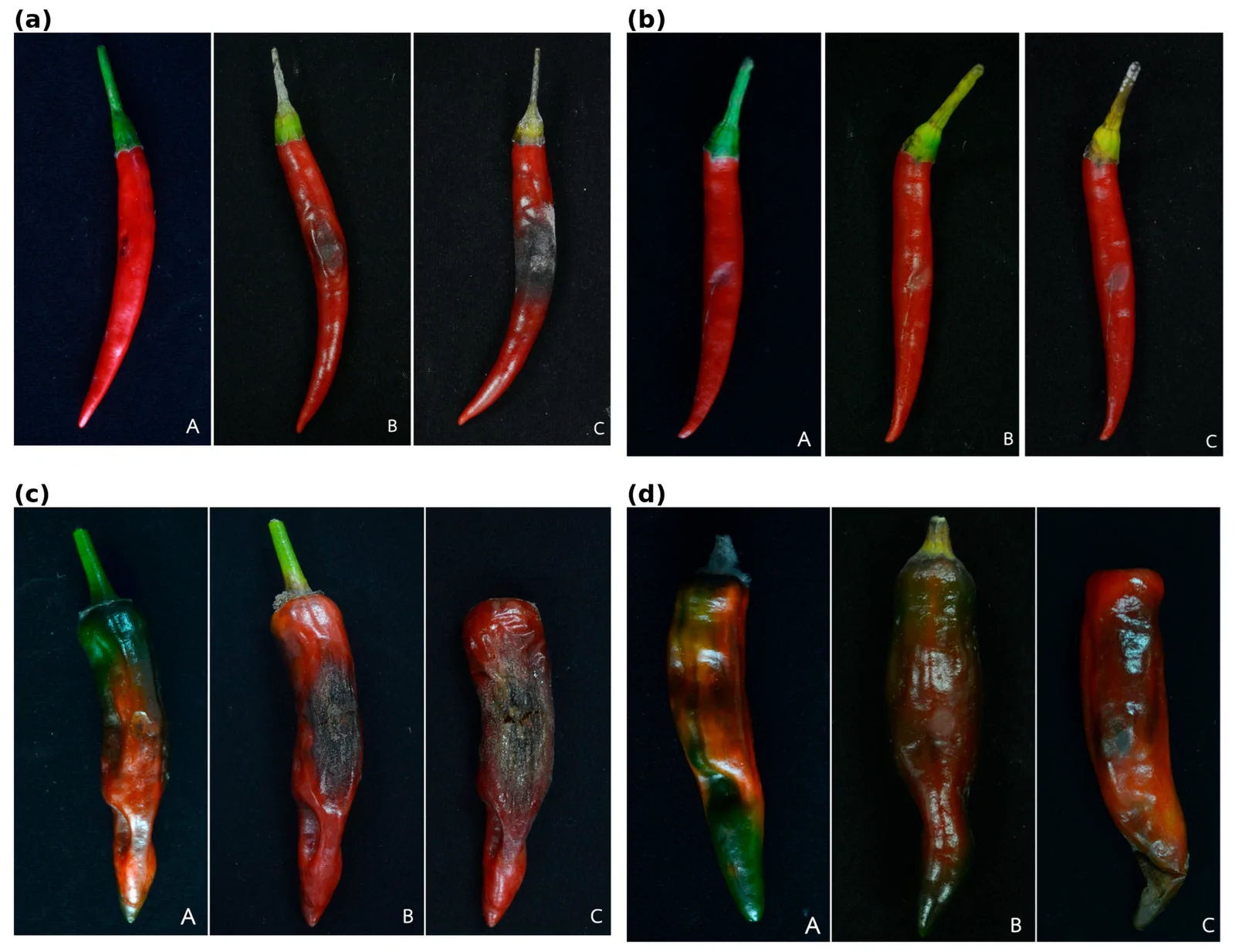
Anthracnose development on detached fruit after non-wound drop inoculation. (**a**) *C. truncatum* KRI015 and (**b**) *C. gloeosporioides* RBR009 on bird’s eye chili; (**c**) KRI015 and (**d**) RBR009 on Chee Fah-type chili. Within each panel, fruit are shown (left to right) at 5, 10 and 15 dpi.

Virulence differed significantly among isolates both between and within morphological groups (F-test, *p* ≤ 0.01; Table 3 and Table 4; Figure 3). On bird’s eye chili fruit, *C. truncatum* isolate KRI015 (Kanchanaburi) produced the highest disease severity scores, 0.4, 4.6 and 6.6 at 5, 10 and 15 days post-inoculation (dpi), respectively, significantly higher than all other isolates at 10 and 15 dpi (DMRT, *p* ≤ 0.05). On Chee Fah-type chili (‘Phrik Man’) fruit, KRI015 reached 0.4, 5.4 and 9.0 at the same time points, attaining the maximum score by 15 dpi. *C. gloeosporioides* isolate RBR009 (Ratchaburi) ranked second, with scores of 0/1.0/3.0 and 0.4/2.2/3.7 on bird’s eye chili and Chee Fah-type chili, respectively. Group I isolates produced low scores (≤0.6 on bird’s eye chili and ≤2.2 on Chee Fah-type chili at 15 dpi). KRI015 and RBR009 were therefore selected as challenge isolates for the PRI experiments.

### 2.4. Efficacy of Plant Resistance Inducers on Fruit

No symptoms were observed on fruit at 7 dpi in any treatment. Disease appeared at 14 dpi and increased markedly by 21 dpi, when control fruit showed 6.50–94.00% disease depending on genotype and isolate (Table 5). At 21 dpi, treatment effects were significant in six of the twelve genotype × isolate combinations: CA365 × CA1163 and CA365 × CA1355 with both isolates, ‘Amphawa Gold’ with KRI015, and CA758 with RBR009 (where the effect was driven by increased disease in CHI-treated fruit). Relative reductions are summarized in Table 6.

On CA365 × CA1163, all inducers significantly reduced disease at 21 dpi. Against KRI015 (control 58.67%), SA (4.13%) and foliar B01 (6.20%) were most effective (93.0% and 89.4% reduction), followed by ASM (7.67%), BABA (9.00%) and CHI (18.00%); soil-drenched B01 (26.33%; 55.1%) was less effective. Against RBR009 (control 51.33%), all treatments reduced disease to 2.33–18.60% (63.8–95.5%) without differing from one another.

On CA365 × CA1355, SA, ASM and CHI reduced disease caused by KRI015 at 14 dpi (75.4%, 65.2% and 39.3%), and SA and ASM remained effective at 21 dpi (69.4% and 52.8%). BABA and foliar B01 did not reduce disease, and foliar B01 had the highest disease at 21 dpi (50.91%). Against RBR009 (control 20.00%), all treatments reduced disease at both time points; SA, CHI and soil-drenched B01 prevented it completely, whereas BABA was least effective (35.7%).

**Figure 4:**
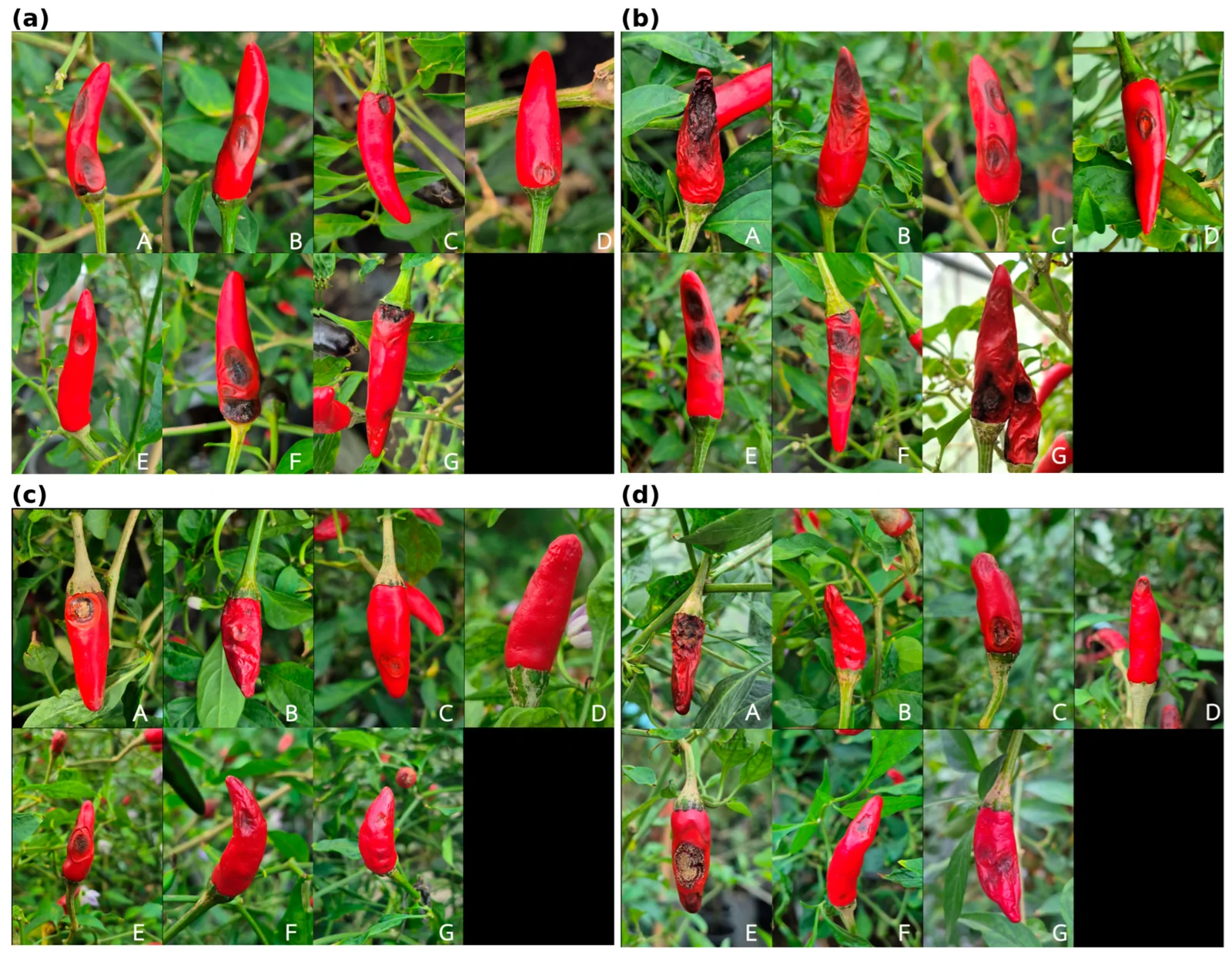
Fruit of CA758 × CA1355 treated with plant resistance inducers and inoculated with (**a**,**b**) *C. truncatum* KRI015 or (**c**,**d**) *C. gloeosporioides* RBR009, at (**a**,**c**) 14 dpi and (**b**,**d**) 21 dpi. Within each panel: A, control; B, SA; C, BABA; D, ASM; E, CHI; F, *B. subtilis* B01 foliar spray; G, *B. subtilis* B01 soil drench.

On the highly susceptible ‘Amphawa Gold’, control fruit inoculated with KRI015 reached 94.00% disease at 21 dpi; all inducers reduced this significantly (46.67–68.67%), but only by 27–50%, with CHI giving the lowest value. With RBR009, differences at 21 dpi were not significant, although foliar B01 (6.67%) and BABA (9.33%) tended to be lowest. On CA758 × CA1163, CA758 × CA1355 and CA758, most differences were not significant; ASM and BABA tended to give low disease with KRI015 on CA758 × CA1355 (1.00% and 10.67% vs. 52.67%; Figure 4) and CA758 (4.00% and 5.00% vs. 50.00%), whereas CHI increased disease on CA758 × CA1163 with KRI015 (50.67% vs. 14.67%) and on CA758 with RBR009 (41.67% vs. 6.50%, *p* ≤ 0.05). Across the five combinations in which inducers significantly reduced disease, SA and ASM significantly reduced disease in all five, and BABA, CHI and each B01 application in four.

### 2.5. Transcriptome Responses to Plant Resistance Inducers

**Figure 5:**
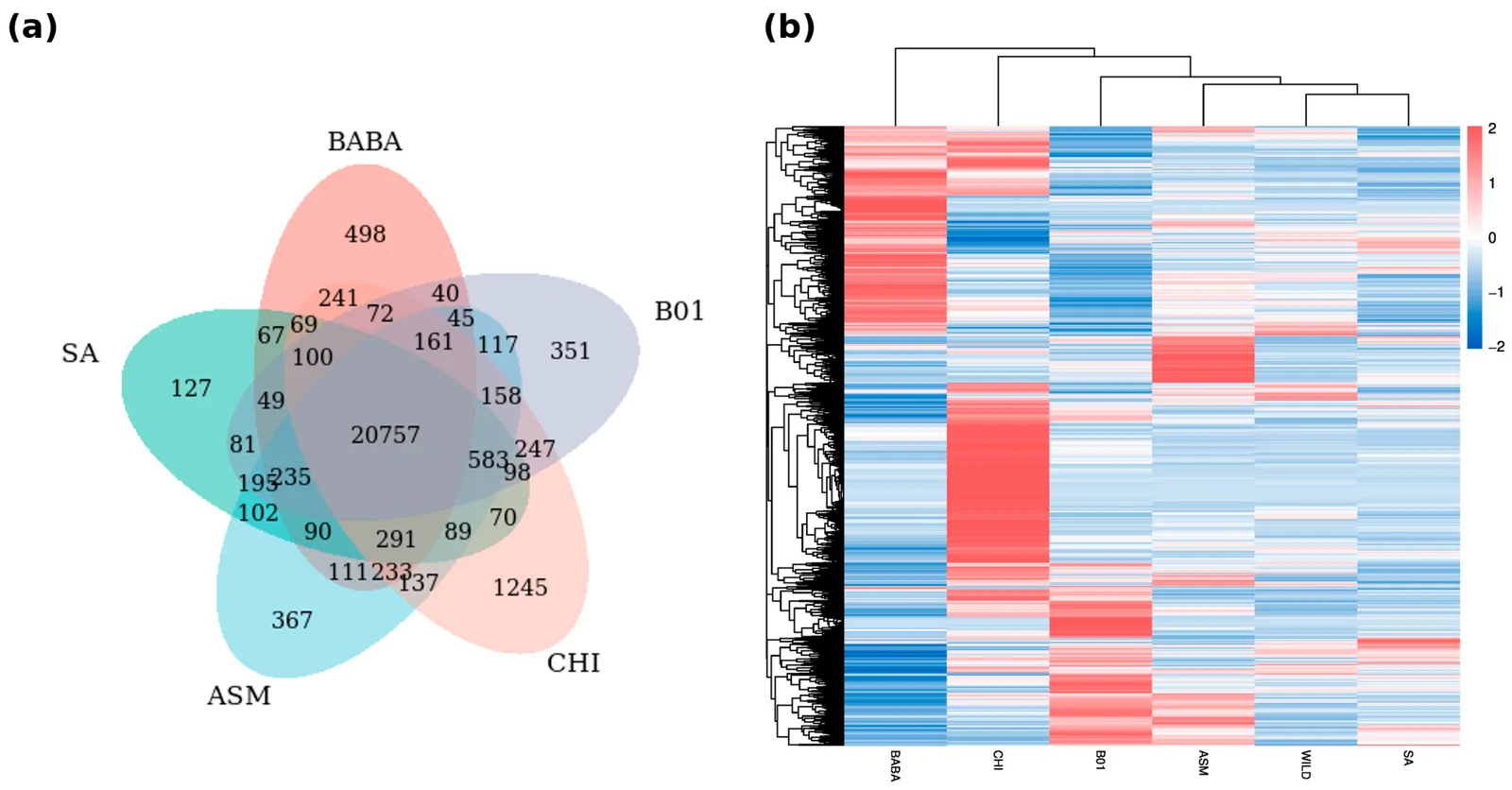
Global transcriptome responses to plant resistance inducers. (**a**) Venn diagram of genes expressed in each treatment; (**b**) hierarchical clustering heatmap of DEG expression across treatments. WILD, untreated control.

To explore the host responses elicited by each inducer, genotype CA758 × CA1355 was sampled 24 h after treatment, without pathogen inoculation, and analyzed by RNA-seq. RNA integrity was high, and the distributions of log_2_(FPKM + 1) were similar among treatments. A core set of 20,757 genes was expressed in all treatments; CHI had the largest number of uniquely expressed genes (1245), followed by BABA (498) and B01 (351) (Figure 5a).

**Figure 6:**
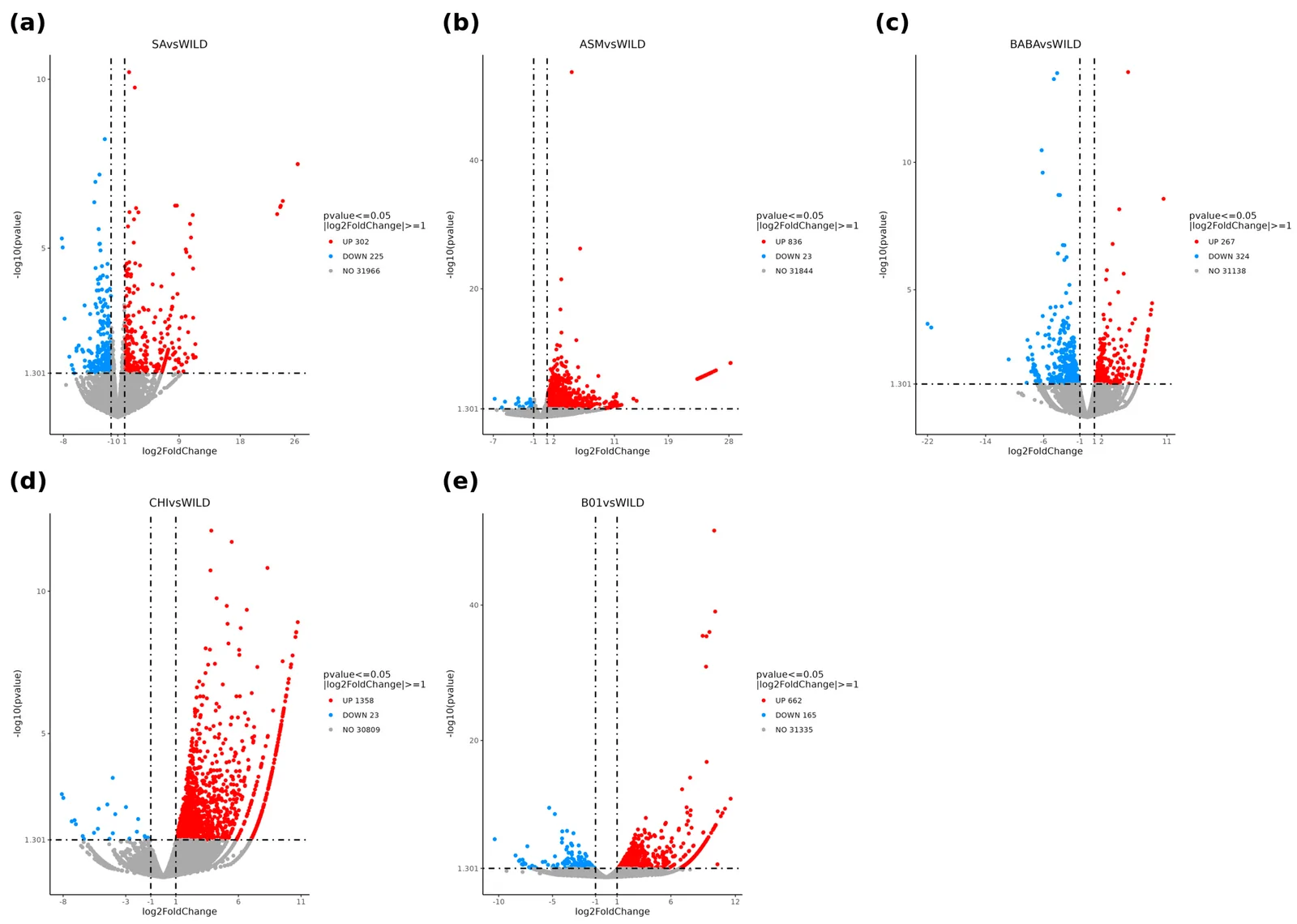
Volcano plots of gene expression in plants treated with (**a**) SA, (**b**) ASM, (**c**) BABA, (**d**) CHI and (**e**) *B. subtilis* B01 relative to the untreated control. Red and blue points denote up- and down-regulated genes (*p* ≤ 0.05, |log_2_FC| ≥ 1).

Using a nominal threshold (*p* ≤ 0.05, |log_2_FC| ≥ 1), CHI produced the most differentially expressed genes (DEGs; 1381, 1358 up-regulated), followed by ASM (859), B01 (827), BABA (591) and SA (527) (Table 7; Figure 5b and Figure 6). BABA was the only treatment with more down- than up-regulated genes. Under the stringent threshold (padj ≤ 0.05), the numbers fell to 280 (CHI), 173 (ASM), 64 (B01), 25 (BABA) and 4 (SA), with the same qualitative pattern: CHI and ASM responses were almost entirely up-regulation, whereas BABA responses were dominated by down-regulation (Table 7).

Gene-level analysis of the stringent DEG set suggested differences in defense-related responses among the inducers (Table 8). ASM up-regulated six genes annotated as 5-epi-aristolochene synthase (EAS; log_2_FC +3.65 to +6.95) and one annotated as cytochrome P450 *CYP71D7* (+4.56), a member of the CYP71D family that includes 5-epi-aristolochene dihydroxylase (EAH). In *Capsicum*, EAS cyclizes farnesyl diphosphate to 5-epi-aristolochene and EAH hydroxylates it to the phytoalexin capsidiol, and both genes are induced by elicitors and by *Colletotrichum* infection of fruit [36,37,38]. These seven genes also had positive fold changes in the other treatments (+1.17 to +4.80; Table 8) but reached padj ≤ 0.05 only with ASM. These genes were therefore induced most strongly by ASM, but their induction was not restricted to ASM. In total, ASM up-regulated 15 terpene synthase genes (+2.28 to +6.95) and the PR-gene transcriptional activator *PTI5* (+2.97). CHI strongly induced genes of the phenylpropanoid and lignin pathways, including *peroxidase 15* (+6.68) and *caffeic acid 3-O-methyltransferase* (+5.49), together with five cell wall/callose-related genes, a chitinase (+3.45) and five protease inhibitor genes (up to +9.55). The capsaicinoid biosynthesis gene *Pun1* was up-regulated by CHI (+3.78), BABA (+2.72) and B01 (+2.71). *Hypersensitivity-related protein 4* was induced by SA, ASM and CHI. In contrast, BABA was the only treatment that significantly repressed direct defense genes (a basic vacuolar glucan endo-1,3-β-glucosidase (−6.26), *endochitinase B* (−3.94) and a defensin (−3.75)), with 17 of its 25 stringent DEGs down-regulated. B01 significantly repressed *salicylate carboxymethyltransferase* (−5.30), which converts SA into methyl salicylate, and induced a protease inhibitor (+10.36), a PR protein and two terpene synthase genes.

ASM also induced the largest number of transcription factor (TF) genes (122), dominated by WRKY (15), bHLH, ERF and MYB/MYB-related (12 each) and NAC (8) families (Table 9). CHI induced 109 TF genes, chiefly MYB/MYB-related (11) and NAC (9); B01 induced 34, with WRKY as the largest family (5); BABA and SA induced 15 and one, respectively.

**Figure 7:**
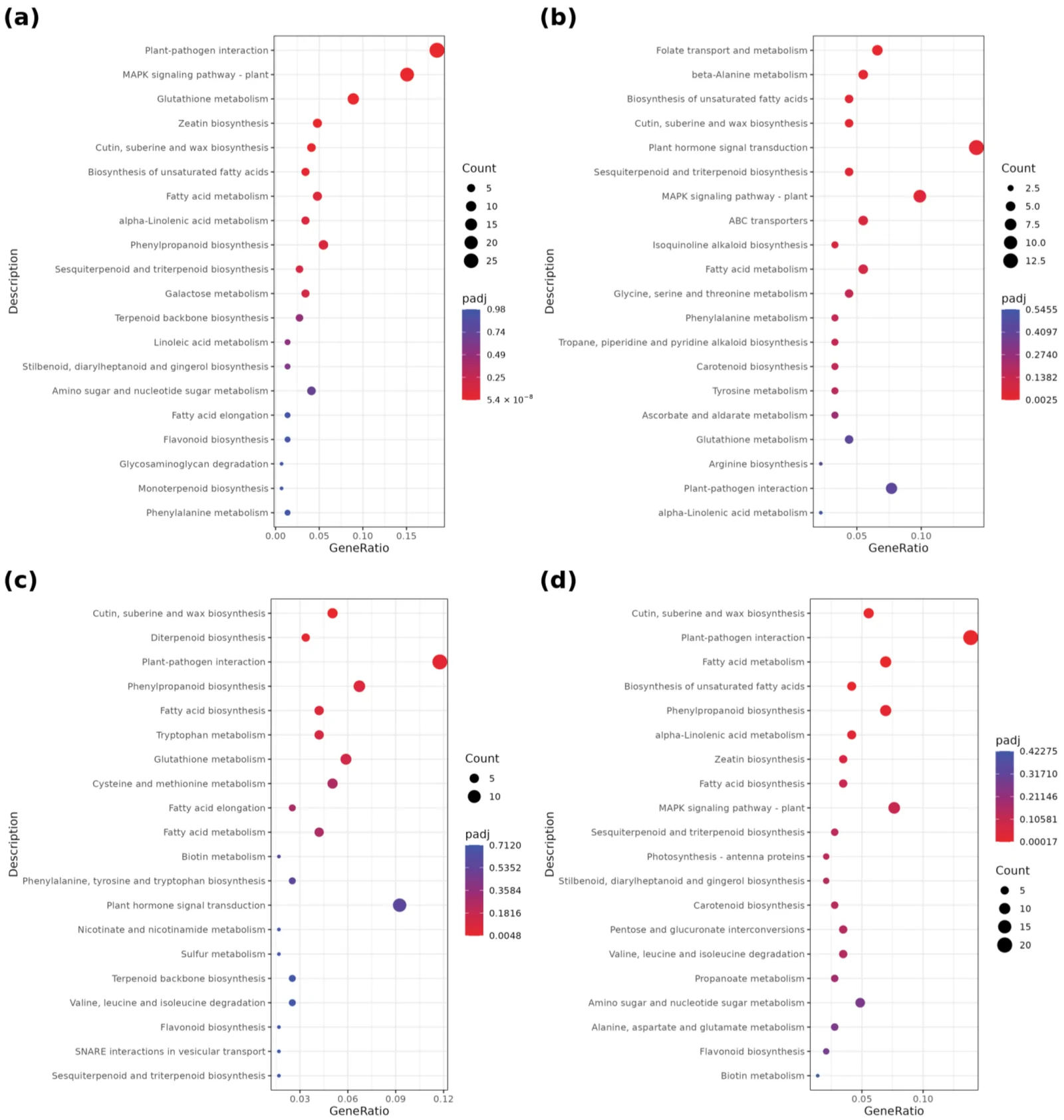
KEGG pathway enrichment of DEGs (*p* ≤ 0.05, |log_2_FC| ≥ 1) in plants treated with (**a**) ASM, (**b**) BABA, (**c**) CHI and (**d**) *B. subtilis* B01. Dot size indicates gene count and color indicates adjusted *p*-value.

Enrichment analyses (nominal DEG lists) supported these patterns (Figure 7; Tables S1 and S2). For ASM, the most significant GO term in the whole data set was *response to fungus* (53 genes, all up-regulated; *p* = 1.1 × 10^−16^), followed by *defense response to fungus* (40 genes, all up-regulated), and the top KEGG pathways were plant–pathogen interaction (27 genes, 26 up), MAPK signaling (22 genes, 21 up) and glutathione metabolism (13 genes, all up). CHI was enriched in cutin, suberine and wax biosynthesis (six genes, all up), diterpenoid biosynthesis and plant–pathogen interaction, and in the GO terms *enzyme inhibitor activity* (21 genes, 20 up) and *cysteine-type endopeptidase inhibitor activity* (eight genes, all up). B01 was enriched in cutin, suberine and wax biosynthesis, plant–pathogen interaction (20 genes, all up), fatty acid metabolism and phenylpropanoid biosynthesis. BABA showed a contrasting pattern in which enriched pathways were mostly down-regulated, including MAPK signaling (nine genes, all down) and plant hormone signal transduction (13 genes, 11 down). The top GO terms for SA, ASM and B01 included nutrient reservoir activity, lipid storage and lipid droplet (Table S1), which are characteristic of seed tissue. Although seeds and placenta were removed before RNA extraction (Section 4.7), small amounts of seed tissue may have remained in some samples; these terms were therefore not interpreted.

### 2.6. Total Phenolic Content

Soluble phenolic content differed significantly among treatments at each sampling time (*p* ≤ 0.01; Table 10). At 24 h, ASM gave the highest content (3.59 mg GAE g^−1^ FW; 31.0% above the control), followed by foliar B01 (3.00; 9.5% above), whereas SA, BABA and CHI were significantly lower than the control. At 48 h, contents declined in all treatments (control 1.24), but all treatments except SA exceeded the control, with CHI most strongly (2.13; +71.8%). At 72 h, foliar B01 (3.19; +42.4%), ASM (2.92; +30.4%) and SA (2.61; +16.5%) exceeded the control, whereas BABA, soil-drenched B01 and CHI were lower. Only ASM (+30.4 to +50.8%) and foliar B01 (+9.5 to +50.0%) were significantly above the control at all three times. At 72 h, foliar B01 gave almost twice the content of soil-drenched B01 (3.19 vs. 1.67).

## 3. Discussion

### 3.1. Colletotrichum Species Complexes Associated with Chili Anthracnose

The 74 isolates recovered from nine provinces fell into three morphological groups, and multilocus analysis confirmed that representative isolates belonged to the *C. gloeosporioides* and *C. truncatum* species complexes, indicating that more than one species complex is associated with anthracnose in Thai chili production, although the sampling was limited in scope. Groups II and III, whose conidial characters correspond to these two complexes, accounted for 44.6% and 39.2% of isolates. Because only seven isolates were sequenced, these values describe morphological groups rather than species complex frequencies. Previous studies identified *C. siamense* (*C. gloeosporioides* species complex), *C. truncatum* and *C. scovillei* (*C. acutatum* species complex) as the main causal agents of chili anthracnose in Thailand and Asia [3,5,6]. The high proportion of Group II isolates differs from earlier reports in which *C. truncatum* predominated; sequencing of more isolates is needed to determine whether this reflects the sampling or a change in the pathogen population.

Two Group I isolates (fusiform conidia) clustered with the *C. gloeosporioides* species complex in the multilocus analysis. Overlapping phenotypes among species complexes limit morphology-based identification [3], and ITS alone lacks resolution within the *C. gloeosporioides* and *C. acutatum* complexes [33,34]. The *ACT* and *CHS-1* loci used here also provide limited species-level resolution, and bootstrap support within the *C. gloeosporioides* clade was low. This result should therefore be interpreted cautiously and confirmed with more informative loci (*ApMat* and *GS* for the *C. gloeosporioides* complex [39], and *GAPDH*, *TUB2* and *HIS3* for the *C. acutatum* complex [34,40]) before species-level conclusions are drawn.

### 3.2. Pathogenicity and Virulence Variation

The high virulence of *C. truncatum* KRI015, which reached the maximum score on Chee Fah-type chili by 15 dpi, agrees with reports that *C. truncatum* is among the most aggressive *Colletotrichum* species on ripe chili fruit [3,7,10]. KRI015 originated from Kanchanaburi, an intensive production area with continuous cultivation, which may impose selection for aggressive pathotypes. The marked variation in virulence within morphological groups—0.6–6.6 within Group III and 0.6–3.0 within Group II on bird’s eye chili at 15 dpi—is consistent with intraspecific genetic diversity and local adaptation reported for *Colletotrichum* populations [41,42]. This variability has practical implications: control programs designed around a single species may be inadequate against mixed populations, and challenge isolates for resistance screening or PRI evaluation should be chosen to represent this range. Group I isolates were the least virulent under non-wound inoculation; because the two sequenced Group I isolates clustered with the *C. gloeosporioides* complex, this result cannot be attributed to the *C. acutatum* complex. The progression of symptoms after 5 dpi reflects the hemibiotrophic lifestyle of *Colletotrichum*, with an initial biotrophic phase followed by necrotrophic growth.

### 3.3. Efficacy of Plant Resistance Inducers Depends on Inducer, Genotype and Pathogen

The absence of symptoms at 7 dpi and the sharp increase between 14 and 21 dpi are consistent with the quiescent, hemibiotrophic infection of *Colletotrichum* on fruit, in which appressoria remain latent until fruit physiology favors colonization [7,43]; the 21 dpi assessment therefore reflects PRI efficacy more clearly. SA and ASM gave the most consistent protection, reducing disease significantly in all five genotype × isolate combinations in which inducers significantly reduced disease, and by 87–94% on CA365 × CA1163. SA is the central SAR signal and ASM is a benzothiadiazole analog that activates PR genes through NPR1 [44,45]; SA has also been shown to protect unripe pepper fruit against *C. gloeosporioides* by suppressing appressorium formation [46]. Their lack of effect on ‘Amphawa Gold’ inoculated with RBR009 (reductions of 25.7% and 0%, not significant) nevertheless suggests that the response to SA-pathway inducers depends on host genotype.

Because only 24 h separated treatment from inoculation and the inducers were applied to the fruit surface, the protection observed cannot be attributed exclusively to induced resistance. Chitosan has direct antifungal and film-forming activity [27,47], and SA treatment of pepper fruit has been associated with suppressed appressorium formation by *C. gloeosporioides* [46], which may involve direct as well as host-mediated effects, whereas ASM has little direct antimicrobial activity and acts mainly through host defense [45].

BABA was effective on several genotypes but failed against KRI015 on CA365 × CA1355. BABA primes both SA-dependent and SA-independent defenses [24,48], which may explain its protection on diverse genotypes, but its efficacy is known to vary with host, pathogen, dose and environment. CHI and B01 gave the most variable results. CHI reduced disease strongly on CA365 × CA1163 (69.3–95.5%) and gave the lowest value against KRI015 on ‘Amphawa Gold’, yet increased disease on CA758 × CA1163 (KRI015) and CA758 (RBR009). Chitosan acts both directly on fungi and as a defense elicitor, and reduced postharvest anthracnose of bell pepper by increasing defense enzymes and phenolics [27,49]; its variable performance here agrees with the general dependence of induced resistance on genotype, environment and concentration [50,51,52]. For B01, foliar spray outperformed soil drench on CA365 × CA1163 with KRI015 (89.4% vs. 55.1%); a similar but non-significant trend was observed on ‘Amphawa Gold’ with RBR009 (86.8% vs. 39.5%), and no difference was found on CA365 × CA1355. *Bacillus* spp. suppress disease by direct antagonism as well as by ISR [53,54]; foliar cells on the fruit surface may act directly on the pathogen, whereas drenching relies on root colonization and systemic signaling.

The higher disease on CHI-treated fruit of CA758 with RBR009 (significant) and CA758 × CA1163 with KRI015 (not significant) cannot be explained with the present data, but several factors may be involved. The biological activity of chitosan depends on its molecular weight, degree of deacetylation, concentration and the pH of the formulation, and chitosan dissolved in dilute acid can be phytotoxic at high doses [47,55]; micro-injury of the fruit surface could facilitate infection. A chitosan film may also alter surface chemistry and wettability, potentially affecting conidial adhesion and appressorium formation, and residual polymer or its degradation products could serve as a nutrient source for the pathogen. Finally, responses to chitosan may depend on host genotype. Because the degree of deacetylation and pH of the formulation were not determined, and phytotoxicity and conidial adhesion were not assessed, these explanations remain hypotheses.

Host susceptibility limited PRI efficacy. On ‘Amphawa Gold’, almost all fruit became diseased with KRI015 (control 94.00%), and inducers reduced severity by only 27–50%, consistent with the partial protection typically provided by induced resistance under high disease pressure [51]. The CA758-derived genotypes generally had lower control disease, and differences among treatments were rarely significant. For highly susceptible cultivars, PRIs alone are unlikely to be sufficient and should be combined with other control measures.

### 3.4. Inducer-Specific Transcriptional Programs

The transcriptome data suggest that the inducers activated different programs. The most prominent response was the strong induction by ASM of putative capsidiol-biosynthesis genes. Capsidiol is a sesquiterpenoid phytoalexin of *Capsicum* that inhibits spore germination and the hyphal growth of many fungi [56]; EAS converts farnesyl diphosphate to 5-epi-aristolochene, the committed step, and CYP71D-type hydroxylases (EAHs) complete its conversion to capsidiol [57]; both steps have been characterized in *Capsicum*, where *CaEAS* and *CaEAH* are induced by elicitors and are strongly up-regulated in fruit challenged by *Colletotrichum* [36,37,38]. Co-induction of both steps, together with WRKY TFs (key regulators of SA-dependent defense and SAR [58]), the PR-gene activator *PTI5*, up-regulation of plant–pathogen interaction, MAPK signaling [59] and glutathione metabolism [60] genes, suggests that ASM activates chili defenses at the transcriptional level, including genes of phytoalexin biosynthesis, consistent with its action as a functional SA analog [22] and with the increased defense enzyme activity and phenolics reported in ASM-treated chili challenged with *C. capsici* [61]. However, capsidiol was not quantified and no gene silencing or mutant analysis was performed, so whether the capsidiol pathway contributes to ASM-induced protection remains to be tested, for example, by measuring capsidiol in treated and inoculated fruit and by virus-induced gene silencing of *CaEAS* and *CaEAH*.

Grouping the enriched GO terms and KEGG pathways by function clarifies the differences among inducers. Direct antifungal responses (*response to fungus*, *defense response to fungus*, monooxygenase activity) were enriched only with ASM, consistent with phytoalexin-based defense [62,63]. Inhibition of pathogen enzymes (*enzyme inhibitor* and *cysteine-type endopeptidase inhibitor activity*) was enriched only with CHI, in line with the role of apoplastic protease inhibitors in defense [64]. Structural reinforcement (cutin, suberine and wax biosynthesis, fatty acid metabolism and phenylpropanoid biosynthesis, which build barriers against appressorial penetration [65,66]) was up-regulated by CHI, B01 and, to a lesser extent, ASM, whereas cell wall-related terms were mostly down-regulated by BABA. Signaling pathways (plant–pathogen interaction, MAPK and α-linolenic acid metabolism, the precursor pathway of jasmonates [67]) were induced by ASM, B01 and CHI but repressed by BABA.

For CHI, induction of *peroxidase 15* and *COMT* in the lignin pathway is consistent with chitosan-induced lignification and cell wall reinforcement [27]. Up-regulation of *Pun1*, the acyltransferase catalyzing the final step of capsaicinoid biosynthesis, by CHI, BABA and B01 is of applied interest because capsaicinoids determine pungency and have antimicrobial activity; capsaicinoid contents should be measured in future trials. B01 produced a moderate response, including WRKY TFs, a PR protein, terpene synthases and strong repression of salicylate carboxymethyltransferase (SAMT), which converts SA into methyl salicylate (MeSA). The effect of this repression on SA signaling is uncertain. Lower SAMT activity could retain more free SA locally, but MeSA is itself a mobile SAR signal [68], and in pepper MeSA production via the CbAR9–CbSAHH module is required for systemic resistance to *Colletotrichum* [69]. Repression of *SAMT* could therefore either enhance local SA signaling or weaken MeSA-mediated systemic signaling. Because SA, MeSA and SA conjugates were not measured, neither outcome can be inferred from transcript data, and the link to *Bacillus*-mediated induced resistance [53,54] remains hypothetical. Direct antagonism of B01 was not tested, so its protective effect could reflect induced resistance, antibiosis or both.

BABA-repressed direct defense genes and signaling pathways in the absence of the pathogen, a pattern compatible with priming, in which defenses are not deployed until challenge [20,21,70]. Priming cannot be confirmed without primed-and-challenged samples, however. Among the BABA-repressed genes were 11 of 13 genes of the plant hormone signal transduction pathway. Auxin and SA signaling are mutually antagonistic: SA restricts pathogen growth partly by repressing auxin signaling [71], and auxin interacts with other phytohormones to shape stress responses [72]. Repression of hormone-signaling genes by BABA could therefore reflect a shift in the growth–defense balance before challenge, but hormone levels were not measured and BABA did not protect against KRI015 on CA365 × CA1355, so this interpretation remains tentative. SA induced almost no stringent DEGs at 24 h despite providing the most consistent protection. Several factors may explain this. First, SA-responsive transcription is rapid and often transient [73], so a single 24 h time point may have missed the main response. Second, exogenous SA is rapidly metabolized by glucosylation, methylation and hydroxylation [74], which can lower the active pool reaching the fruit, whereas ASM is a stable functional analog that acts downstream of SA accumulation [22,44]. Third, part of the SA response is NPR1-independent and involves early, low-amplitude changes that may not pass a stringent threshold [16,73]. Because SA levels were not measured in treated fruit, these explanations cannot be distinguished. Because almost none of the genes in the SA-enriched pathways remained significant after multiple-testing correction, SA enrichment results were not interpreted. The number of DEGs is therefore not a proxy for protective efficacy.

Phenolic content was measured in fruit of the same genotype at 24, 48 and 72 h, whereas the transcriptome was profiled only at 24 h, so the two data sets can be compared directly only at 24 h. At 24 h, ASM gave the highest soluble phenolic content, in line with its up-regulation of phenylpropanoid-related genes at the same time point and with reports that ASM increases PAL activity and phenolics in chili [61]; foliar B01 also increased soluble phenolics at all three times. BABA lowered phenolics at 24 and 72 h, consistent with the absence of defense activation before challenge. CHI increased soluble phenolics only at 48 h despite strong induction of lignin-pathway genes at 24 h. One possible explanation is that soluble phenolics were converted into cell wall-bound forms, because the Folin–Ciocalteu assay of methanol extracts measures only soluble phenolics [75,76] and peroxidases consume soluble phenolics during lignification [77]; however, bound phenolics and lignin were not measured, so this remains speculative. Control values also fluctuated (2.74, 1.24 and 2.24 mg GAE g^−1^ at 24, 48 and 72 h) even though all samples were collected at the same time of day and fruit stage (Section 4.8); treatments were therefore compared only within each sampling time.

### 3.5. Limitations and Future Work

This study has several limitations. First, RNA-seq was performed on fruit tissue at a single time point (24 h) without pathogen challenge, so it cannot resolve which cell types (e.g., epidermis, pericarp parenchyma or vascular tissue) drive the cuticle, lignin and phytoalexin responses, or how these responses change after infection; single-cell and spatial transcriptomics can resolve such cell-type-specific responses [78]. Second, enrichment analyses used nominal DEG lists, and hormone, capsidiol and bound-phenolic levels were not measured. Third, direct effects of the inducers on the pathogen were not tested. Future work should combine primed-and-challenged time courses, RT-qPCR validation, metabolite and hormone quantification, and in vitro assays with the pathogen.

## 4. Materials and Methods

### 4.1. Survey, Sample Collection and Isolation

Chili fruit showing typical anthracnose symptoms (sunken, water-soaked lesions with or without acervuli) were collected between October 2024 and March 2025 from nine provinces—Nakhon Pathom, Kanchanaburi, Pathum Thani, Samut Sakhon, Phetchabun, Nakhon Ratchasima, Prachuap Khiri Khan, Ratchaburi and Tak—targeting commercial fields with a history of continuous cultivation and chronic anthracnose. Isolation followed the tissue transplanting technique [79]: tissue pieces (5 × 5 mm) from the lesion margin were surface-sterilized in 2% NaOCl for 2 min, rinsed three times in sterile distilled water, blotted dry and placed on PDA (HiMedia, Mumbai, India) at 25–30 °C for 2–3 days. Pure cultures were obtained by single-spore isolation on water agar and maintained on PDA under a 12 h photoperiod (fluorescent light) at 25–30 °C.

### 4.2. Morphological Identification

Isolates were grown on PDA for 7 days, and colony color, texture and the presence of conidial masses were recorded. Conidial shape and dimensions and the presence of setae and appressoria were examined at 1000× magnification. Isolates were grouped by colony and conidial characters, which were compared with descriptions of *Colletotrichum* species complexes [33,34,35]; species complex membership was determined only by multilocus analysis (Section 4.3).

### 4.3. Molecular Identification and Phylogenetic Analysis

Seven representative isolates (KRI002, KRI015, NMA003, PKN002, PNB001, RBR002 and RBR009) were analyzed; they were chosen to cover the three morphological groups, five provinces and the range of virulence in the pathogenicity test. Mycelium from 7-day-old potato dextrose broth cultures was ground in liquid nitrogen, and genomic DNA was extracted using the GF-1 Fungus DNA Extraction Kit (Vivantis Technologies, Subang Jaya, Malaysia). DNA quality was checked by 1.3% agarose gel electrophoresis and a NanoPhotometer (Implen, Westlake Village, CA, USA). ITS, *ACT* and *CHS-1* were amplified with the primers in Table 11. PCR contained 1× *Taq* buffer, 2.5 mM MgCl_2_, 1 U *Taq* polymerase (Thermo Scientific, Vilnius, Lithuania), 0.2 mM dNTPs, 0.5 µM of each primer and 10–25 ng of DNA, with 35 cycles of 95 °C for 30 s, locus-specific annealing for 1 min and 72 °C for 1 min. Amplicons were purified with a FavorPrep GEL/PCR Purification Mini Kit (Favorgen, Ping Tung, Taiwan) and sequenced directly (SolGent Co., Ltd., Daejeon, Korea). Sequences were compared with GenBank using BLASTn (https://blast.ncbi.nlm.nih.gov), and neighbor-joining trees (1000 bootstrap replicates) were constructed for each locus and for the concatenated data set in MEGA version X [80], with *Leptosphaeria veronicae* CBS 145.84 as the outgroup and *C. truncatum* CBS 151.35, *C. gloeosporioides* IMI 356878 and *C. acutatum* CBS 144.29 as references. No reference culture was grown alongside the isolates; published GenBank sequences of these reference strains served as the references (positive controls) in the phylogenetic analysis.

### 4.4. Pathogenicity Test

Ripe fruit of large-fruited bird’s eye chili and Chee Fah-type chili (‘Phrik Man’) were washed with sterile distilled water and placed on wire mesh in sealed plastic boxes containing sterile water to maintain humidity. Conidial suspensions (10^6^ conidia mL^−1^) prepared from 7-day-old PDA cultures were drop-inoculated (10 µL) onto the center of unwounded fruit, and boxes were incubated at 25–30 °C. Disease severity was scored at 5, 10 and 15 dpi on a 0–9 scale modified from Montri et al. [5]: 0, no symptoms; 1, lesion covering 1–2% of the fruit surface; 3, >2–5%; 5, >5–15%; 7, >15–25% with necrosis; 9, >25% with necrosis and acervuli.

### 4.5. Plant Material and Plant Resistance Inducers

Five PRIs were evaluated: SA (Sigma-Aldrich, Saint–Quentin–Fallavier, France), ASM (Ciba–Geigy, Basel, Switzerland) and BABA (Sigma-Aldrich, Taufkirchen, Germany), each at 0.5 mM; CHI (KTF, Kanchanaburi, Thailand) at 1000 µL L^−1^; and *B. subtilis* B01 (Department of Plant Pathology, Faculty of Agriculture at Kamphaeng Saen, Kasetsart University, Nakhon Pathom, Thailand) at 10^7^ CFU mL^−1^. B01 was cultured for 24 h, pelleted by centrifugation and resuspended in sterile distilled water [84]. Six genotypes were used: four F_1_ hybrids (CA365 × CA1163, CA365 × CA1355, CA758 × CA1163 and CA758 × CA1355) and two commercial cultivars (CA758 and the large-fruited bird’s eye chili ‘Amphawa Gold’). These concentrations were selected on the basis of a dose–response screen (0.25, 0.5 and 1.0 mM for SA, ASM and BABA; 500, 1000 and 1500 µL L^−1^ for CHI) on pepper (*Capsicum annuum*) seedlings in our previous study with the same products and B01 strain [85], and lie within the ranges commonly used for these inducers in Solanaceae [11]. CHI was a commercial formulation of shrimp-derived chitosan. B01 was identified as *B. subtilis* with the VITEK 2 system and a BCL card (bioMérieux, Durham, NC, USA) [86].

### 4.6. Fruit Assay

Greenhouse-grown plants at the fruiting stage were sprayed with the abiotic PRIs, or treated with B01 as a foliar spray or soil drench (20 mL per plant), 24 h before spray inoculation of fruit with KRI015 or RBR009 (10^6^ conidia mL^−1^); water-treated plants served as controls. Plants were bagged overnight after inoculation, and the percentage of anthracnose on fruit was assessed at 7, 14 and 21 dpi. The experiment followed a completely randomized design with five replicates.

### 4.7. RNA Sequencing and Transcriptome Analysis

Plants of genotype CA758 × CA1355 were sampled 24 h after PRI treatment without inoculation; untreated plants served as the control (WILD). Each biological replicate consisted of pericarp tissue pooled from five fruits. Tissue (100 mg of pericarp, with seeds and placenta removed) was ground in liquid nitrogen, and total RNA was extracted with the RNeasy Plant Mini Kit (Qiagen, Hilden, Germany) including on-column DNase I digestion. RNA purity was checked with a NanoPhotometer (Implen, Westlake Village, CA, USA) and integrity with an RNA Nano 6000 Assay Kit on a Bioanalyzer 2100 (Agilent Technologies, Santa Clara, CA, USA). Libraries were sequenced on an Illumina platform (paired-end; Novogene Co., Ltd., Beijing, China). Reads were quality-filtered with fastp [87] and aligned to the *C. annuum* reference genome (Ensembl Plants release 56; ASM51225v2) with HISAT2 (version 2.2.1) [88]. Differential expression was analyzed with DESeq2 (version 1.42.0) [89] in a model including all samples. DEGs were defined at padj ≤ 0.05 and |log_2_FC| ≥ 1 for gene-level inference; a nominal threshold (*p* ≤ 0.05, |log_2_FC| ≥ 1) was used to generate gene lists for GO and KEGG enrichment. Transcription factors were classified by family.

### 4.8. Total Phenolic Content

Soluble phenolic content was determined by the Folin–Ciocalteu method [90,91] in fruit of genotype CA758 × CA1355 sampled 24, 48 and 72 h after PRI treatment (three replicates). Ground tissue (0.1 g) was extracted with 10 mL of 80% methanol and centrifuged at 12,000× *g* and 4 °C for 5 min. The supernatant (1 mL) was mixed with 1 mL of Folin–Ciocalteu reagent (Merck, Darmstadt, Germany) for 10 min, followed by 10 mL of 7% Na_2_CO_3_ and 13 mL of distilled water, and incubated in the dark for 90 min. Absorbance was read at 750 nm (S-22 UV/Vis spectrophotometer; BOECO, Hamburg, Germany), and results were expressed as mg gallic acid equivalent (GAE) g^−1^ fresh weight. All samples were collected between 15:00 and 16:00 from fruit at the mature-green stage.

### 4.9. Statistical Analysis

Data were subjected to analysis of variance, and means were separated by Duncan’s multiple range test at *p* ≤ 0.05 [92] using R version 4.5.1 (R Core Team, Vienna, Austria) with the agricolae package in RStudio 2025.05.1+513 (Posit Software, Boston, MA, USA).

## 5. Conclusions

Isolates from anthracnose-affected chili in Thailand fell into three morphological groups, and representative isolates belonged to the *C. gloeosporioides* and *C. truncatum* species complexes, with marked virulence variation within groups; *C. truncatum* KRI015 was the most aggressive isolate. On fruit, SA and ASM provided the most consistent protection across genotypes, whereas BABA, CHI and *B. subtilis* B01 were genotype-, pathogen- and application-dependent, and a highly susceptible cultivar was only partly protected. The transcriptome data suggest that the inducers elicited distinct programs: induction of putative capsidiol-biosynthesis genes, WRKY transcription factor genes and phenolic accumulation for ASM; structural and cuticular defenses and protease inhibitors for CHI; repression of SAMT by B01 and phenolic accumulation with foliar B01; and a repression pattern compatible with priming for BABA. These results identify SA and ASM as the leading candidates, with ASM showing the clearest transcriptional signature, for integrating PRIs into anthracnose management with reduced fungicide use. Whether these transcriptional programs cause the protection observed remains to be tested.

## Figures and Tables

**Table 1:** Sampling locations, chili types and number of *Colletotrichum* isolates per province.

Province	District	Sub-District	Chili Type	Isolate Code	Isolates, *n* (%)
Kanchanaburi	Tha Maka	Pong Tuek	Daeng Jinda	KRI	24 (32.4)
Kanchanaburi	Lao Khwan	Lao Khwan	Bird’s eye chili		
Kanchanaburi	Lao Khwan	Nong Pradu	Bird’s eye chili		
Tak	Phop Phra	Ruam Thai Phatthana	Bird’s eye chili	TAK	4 (5.4)
Nakhon Pathom	Mueang	Phra Prathon	Bird’s eye chili	NPT	10 (13.5)
Nakhon Pathom	Mueang	Wang Taku	Daeng Jinda		
Nakhon Pathom	Kamphaeng Saen	Kamphaeng Saen	Bird’s eye chili; sweet pepper		
Nakhon Ratchasima	Nong Bun Mak	Saraphi	Bird’s eye chili	NMA	9 (12.2)
Nakhon Ratchasima	Non Sung	Kham Thao	Bird’s eye chili		
Pathum Thani	Nong Suea	Bueng Ka Sam	Small bird’s eye chili	PTE	3 (4.1)
Prachuap Khiri Khan	Thap Sakae	Ang Thong	Bird’s eye chili; Kra-riang	PKN	8 (10.8)
Phetchabun	Khao Kho	Khao Kho	Daeng Jinda	PNB	4 (5.4)
Ratchaburi	Suan Phueng	Suan Phueng	Bird’s eye chili	RBR	9 (12.2)
Samut Sakhon	Mueang	Tha Sai	Bird’s eye chili	SKN	3 (4.1)
Total					74 (100.0)

**Table 2:** Morphological groups of *Colletotrichum* isolates from chili in Thailand.

Morphological Group	No. of Isolates	% of Total	Conidial Morphology
Group I (fusiform conidia)	12	16.2	Fusiform, acute ends; 12–18 × 3–6 µm
Group II (cylindrical conidia)	33	44.6	Cylindrical to ellipsoidal; 12–20 × 4–6 µm
Group III (falcate conidia)	29	39.2	Falcate, truncate base; 18–27 × 2.5–6 µm
Total	74	100.0	

Group I, II and III characters resemble those of the *C. acutatum*, *C. gloeosporioides* and *C. truncatum* species complexes, respectively; species complex membership was confirmed only for the seven sequenced isolates (Figure 2).

**Table 3:** Anthracnose severity on bird’s eye chili fruit inoculated with *Colletotrichum* isolates at 5, 10 and 15 days post-inoculation (dpi).

Morphological Group	Isolate	Disease Severity Score ^1^		
		5 dpi	10 dpi	15 dpi
Group I	NMA009	0 c	0 e	0 e
	PKN002 ^2^	0 c	0 e	0.4 e
	PNB001 ^2^	0 c	0 e	0.2 e
	RBR008	0 c	0 e	0.6 e
	TAK004	0 c	0 e	0.4 e
Group II	KRI002	0.8 a	1.2 bc	1.4 cde
	KRI017	0 c	0.4 cde	0.6 e
	NMA003	0 c	0.4 cde	0.6 e
	RBR002	0.2 bc	0.4 cde	0.8 de
	RBR009	0 c	1.0 bcd	3.0 b
Group III	KRI012	0 c	1.4 b	2.3 bc
	KRI015	0.4 b	4.6 a	6.6 a
	KRI023	0 c	1.5 b	2.0 bcd
	RBR001	0 c	0.2 de	0.6 e
	SKN002	0 c	0 e	0.6 e
F-test		**	**	**

^1^ 0–9 scale (Section 4.4). Means within a column followed by the same letter are not significantly different (DMRT, *p* ≤ 0.05); ** *p* ≤ 0.01. ^2^ Clustered with the *C. gloeosporioides* species complex in the multilocus analysis (Figure 2). Ten fruit per isolate.

**Table 4:** Anthracnose severity on Chee Fah-type chili (‘Phrik Man’) fruit inoculated with *Colletotrichum* isolates at 5, 10 and 15 dpi.

Morphological Group	Isolate	Disease Severity Score ^1^		
		5 dpi	10 dpi	15 dpi
Group I	NMA009	0 b	0 c	2.2 bcde
	PKN002 ^2^	0 b	0 c	0.6 ef
	PNB001 ^2^	0 b	0.4 c	0.8 def
	RBR008	0 b	0.2 c	0.8 def
	TAK004	0 b	0.4 c	1.0 def
Group II	KRI002	0.6 a	0 c	3.0 bc
	KRI017	0 b	0.4 c	1.2 cdef
	NMA003	0 b	0 c	0.6 ef
	RBR002	0 b	0.6 c	0.6 ef
	RBR009	0.4 a	2.2 b	3.7 b
Group III	KRI012	0 b	0.5 c	2.6 bcd
	KRI015	0.4 a	5.4 a	9.0 a
	KRI023	0 b	0 c	0 f
	RBR001	0 b	0 c	0.6 ef
	SKN002	0 b	0.2 c	0.6 ef
F-test		**	**	**

^1^ 0–9 scale (Section 4.4). Means within a column followed by the same letter are not significantly different (DMRT, *p* ≤ 0.05); ** *p* ≤ 0.01. ^2^ Clustered with the *C. gloeosporioides* species complex in the multilocus analysis (Figure 2). Ten fruit per isolate.

**Table 5:** Anthracnose (%) on fruit of six chili genotypes treated with plant resistance inducers 24 h before spray inoculation with *C. truncatum* KRI015 or *C. gloeosporioides* RBR009.

Treatment	KRI015		RBR009	
	14 dpi	21 dpi	14 dpi	21 dpi
CA365 × CA1163				
Control	13.13	58.67 a	4.80	51.33 a
SA	0.40	4.13 c	0.00	4.47 b
ASM	0.00	7.67 bc	0.00	3.00 b
BABA	1.37	9.00 bc	1.67	6.00 b
CHI	1.00	18.00 bc	0.00	2.33 b
B01 (spray)	6.47	6.20 c	0.33	10.47 b
B01 (drench)	4.67	26.33 b	8.67	18.60 b
F-test	ns	**	ns	**
CA365 × CA1355				
Control	30.34 a	30.34 ab	20.00 a	20.00 a
SA	7.47 b	9.27 c	0.00 c	0.00 c
ASM	10.56 b	14.32 c	0.00 c	5.17 c
BABA	30.73 a	33.80 a	12.87 b	12.87 b
CHI	18.43 b	18.43 bc	0.00 c	0.00 c
B01 (spray)	38.04 a	50.91 a	2.59 c	5.17 c
B01 (drench)	15.45 bc	30.91 bc	0.00 c	0.00 c
F-test	**	**	**	**
CA758 × CA1163				
Control	3.00	14.67	8.00	29.33
SA	0.00	10.67	0.00	0.00
ASM	0.33	10.67	2.00	8.00
BABA	2.00	13.67	0.00	7.53
CHI	9.67	50.67	0.13	0.67
B01 (spray)	4.00	29.00	6.00	11.67
B01 (drench)	5.50	22.00	1.67	10.67
F-test	ns	ns	ns	ns
CA758 × CA1355				
Control	18.00 a	52.67	9.47	16.00
SA	2.47 b	22.67	0.67	1.33
ASM	0.33 b	1.00	0.13	2.67
BABA	0.67 b	10.67	0.50	11.00
CHI	2.67 b	21.33	4.87	13.00
B01 (spray)	5.87 b	38.67	1.00	7.00
B01 (drench)	5.00 b	40.67	0.93	7.00
F-test	**	ns	ns	ns
CA758				
Control	11.67	50.00	3.00 b	6.50 b
SA	0.70	29.00	0.00 b	0.00 b
ASM	0.00	4.00	1.47 b	4.33 b
BABA	0.67	5.00	0.47 b	6.67 b
CHI	2.00	17.00	25.67 a	41.67 a
B01 (spray)	6.00	51.33	0.00 b	5.33 b
B01 (drench)	2.00	18.67	2.33 b	4.67 b
F-test	ns	ns	*	*
Amphawa Gold				
Control	53.00	94.00 a	1.00 a	50.67
SA	20.67	68.67 b	0.33 ab	37.67
ASM	6.00	54.00 b	0.67 ab	50.67
BABA	7.33	54.00 b	0.00 b	9.33
CHI	6.00	46.67 b	0.00 b	23.33
B01 (spray)	9.67	68.33 b	0.00 b	6.67
B01 (drench)	8.67	64.67 b	0.33 ab	30.67
F-test	ns	*	*	ns

Means of five replicates. Within each genotype, means in a column followed by the same letter are not significantly different (DMRT, *p* ≤ 0.05); ns, not significant; * *p* ≤ 0.05; ** *p* ≤ 0.01. SA, salicylic acid; ASM, acibenzolar-S-methyl; BABA, β-aminobutyric acid; CHI, chitosan; B01, *Bacillus subtilis* B01.

**Table 6:** Reduction (%) in anthracnose relative to the control at 21 dpi (KRI015/RBR009).

Genotype	SA	ASM	BABA	CHI	B01 Spray	B01 Drench
CA365 × CA1163	93/91	87/94	85/88	69/95	89/80	55/64
CA365 × CA1355	69/100	53/74	−11/36	39/100	−68/74	−2/100
CA758 × CA1163	27/100	27/73	7/74	−245/98	−98/60	−50/64
CA758 × CA1355	57/92	98/83	80/31	60/19	27/56	23/56
CA758	42/100	92/33	90/−3	66/−541	−3/18	63/28
Amphawa Gold	27/26	43/0	43/82	50/54	27/87	31/39

Calculated as (control − treatment)/control × 100 from the means in Table 5; negative values indicate more disease than in the control. Significance should be read from Table 5: at 21 dpi, differences were not significant for CA758 × CA1163 (both isolates), CA758 × CA1355 (both), CA758 (KRI015) or ‘Amphawa Gold’ (RBR009).

**Table 7:** Differentially expressed genes (DEGs) in chili genotype CA758 × CA1355 24 h after treatment with plant resistance inducers, relative to the untreated control.

Treatment	*p* ≤ 0.05, |log_2_FC| ≥ 1 ^1^			padj ≤ 0.05 ^2^	
	Total	Up	Down	Total	Up/Down
SA	527	302	225	4	n.r.
ASM	859	836	23	173	172/1
BABA	591	267	324	25	8/17
CHI	1381	1358	23	280	279/1
B01	827	662	165	64	55/9

^1^ Nominal threshold; gene lists used for transcription factor classification and the enrichment analyses (Figure 7; Tables S1 and S2). ^2^ Benjamini–Hochberg-adjusted threshold used for gene-level inference (Table 8); n.r., not reported. B01, *Bacillus subtilis* B01.

**Table 8:** Expression (log_2_ fold change relative to the untreated control) of selected defense-related genes.

Gene ID	Annotation	SA	ASM	BABA	CHI	B01
T459_04880	5-epi-aristolochene synthase	+1.81	+3.85 *	+1.56	+1.77	+3.00
T459_29603	5-epi-aristolochene synthase	+3.79	+6.95 *	+4.26	+4.79	+4.80
T459_31825	5-epi-aristolochene synthase	+2.11	+3.65 *	+1.85	+2.23	+3.10
T459_31831	5-epi-aristolochene synthase	+2.01	+4.93 *	+1.30	+1.17	+2.89
T459_31827	5-epi-aristolochene synthase	+2.14	+4.54 *	+1.23	+2.32	+2.61
T459_35185	5-epi-aristolochene synthase	+2.17	+4.39 *	+1.89	+1.65	+2.62
T459_29602	Cytochrome P450 71D7	+1.82	+4.56 *	+2.54	+2.26	+2.72
T459_12845	Peroxidase 15	+1.71	+2.54	+4.69	+6.68 *	+2.73
T459_18427	Caffeic acid 3-O-methyltransferase	+1.92	−0.46	+2.72	+5.49 *	+1.94
T459_06020	*Pun1* acyltransferase	+1.38	−0.51	+2.72 *	+3.78 *	+2.71 *
novel.5079	Hypersensitivity-related protein 4	+3.03 *	+3.13 *	+1.82	+5.09 *	+1.61
T459_05079	*PTI5* (PR-gene transcriptional activator)	+1.44	+2.97 *	+0.42	+1.23	+1.06
T459_13623	Glucan endo-1,3-β-glucosidase	−0.32	+0.13	−6.26 *	−0.67	+0.17
T459_33510	Endochitinase B	−2.03	−0.94	−3.94 *	−0.34	−0.29
novel.7445	Defensin	−0.28	+0.29	−3.75 *	+1.16	+1.38
T459_07530	Salicylate carboxymethyltransferase	−1.02	−0.88	−1.44	−0.71	−5.30 *

* padj ≤ 0.05; values without an asterisk were not significant.

**Table 9:** Transcription factor genes among DEGs (*p* ≤ 0.05, |log_2_FC| ≥ 1), by family.

Treatment	Total TFs	WRKY	NAC	ERF	bHLH	MYB/MYB-Related
SA	1	0	1	0	0	0
ASM	122	15	8	12	12	12
BABA	15	0	0	0	0	1
CHI	109	2	9	7	8	11
B01	34	5	2	2	3	4

**Table 10:** Soluble phenolic content (mg gallic acid equivalents g^−1^ fresh weight) in chili tissue after treatment with plant resistance inducers.

Treatment	24 h	48 h	72 h
Control	2.74 c	1.24 d	2.24 d
SA	2.25 d	0.99 e	2.61 c
ASM	3.59 a	1.87 b	2.92 b
BABA	2.22 d	1.49 c	1.75 e
CHI	2.46 d	2.13 a	1.56 e
B01 (foliar spray)	3.00 b	1.86 b	3.19 a
B01 (soil drench)	2.88 bc	1.80 b	1.67 e
F-test	**	**	**
CV (%)	4.87	8.61	5.28

Means within a column followed by the same letter are not significantly different (DMRT, *p* ≤ 0.05); ** *p* ≤ 0.01.

**Table 11:** Primers used for molecular identification of *Colletotrichum* spp.

Locus	Primer	Sequence (5′–3′)	Ta (°C)	Reference
ITS	ITS5	GGAAGTAAAAGTCGTAACAAGG	56	[81]
	ITS4	TCCTCCGCTTATTGATATGC		
*ACT*	ACT-512F	ATGTGCAAGGCCGGTTTCGC	58	[82]
	ACT-783R	TACGAGTCCTTCTGGCCCAT		
*CHS-1*	CHS-79F	TGGGGCAAGGATGCTTGGAAGAAG	58	[82]
	CHS-345R	TGGAAGAACCATCTGTGAGAGTTG		
*TUB2* ^1^	T1	AACATGCGTGAGATTGTAAGT	55	[83]
	T2	TAGTGACCCTTGGCCCAGTTG		

^1 ^*TUB2* primers are listed for reference; *TUB2* was not included in the present phylogeny.

## Data Availability

The original contributions presented in this study are included in the article/Supplementary Material. Further inquiries can be directed to the corresponding author.

## Supplementary Materials

The following supporting information can be downloaded at https://www.mdpi.com/article/10.3390/plants15213257/s1, Table S1: Five most significant GO terms for each treatment; Table S2: Significantly enriched KEGG pathways for each treatment.
