# Supplementary material for: Plant Resistance Inducers Suppress Anthracnose on Chili Fruit and Are Associated with Inducer-Specific Transcriptional Responses
Source: Plants (Basel). 2026 Oct 22;15(21):3257. doi: 10.3390/plants15213257 (PMC13765383; doi:10.3390/plants15213257)
Supplement: Supplementary file 1 [file plants-15-03257-s001.zip › plants-4622828-supplementary.pdf]

Supplementary Materials — Plant Resistance Inducers Suppress Anthracnose on Chili Fruit and Are Associated with Inducer-Specific Transcriptional Responses

Table S1. Five most significant GO terms for each treatment.

Treatment	Category	GO ID	Description	Gene ratio	p-value	Up/Down
SA	MF	GO:0045735	nutrient reservoir activity	16/326	1.4×10^{-14}	15/1
SA	CC	GO:0005576	extracellular region	34/220	1.0×10^{-13}	10/24
SA	CC	GO:0012511	monolayer-surrounded lipid storage body	8/220	1.3×10^{-12}	8/0
SA	BP	GO:0019915	lipid storage	8/273	7.5×10^{-11}	8/0
SA	CC	GO:0005811	lipid droplet	9/220	3.0×10^{-10}	8/1
ASM	BP	GO:0009620	response to fungus	53/462	1.1×10^{-16}	53/0
ASM	MF	GO:0045735	nutrient reservoir activity	18/549	2.1×10^{-13}	18/0
ASM	BP	GO:0050832	defense response to fungus	40/462	6.2×10^{-12}	40/0
ASM	CC	GO:0012511	monolayer-surrounded lipid storage body	8/405	1.5×10^{-10}	8/0
ASM	MF	GO:0004497	monooxygenase activity	38/549	9.9×10^{-10}	36/2
BABA	CC	GO:0005576	extracellular region	36/224	2.5×10^{-15}	16/20
BABA	CC	GO:0005615	extracellular space	11/224	3.5×10^{-8}	1/10
BABA	MF	GO:0046527	glucosyltransferase activity	11/286	6.9×10^{-7}	1/10
BABA	MF	GO:0016762	xyloglucan:xyloglucosyl transferase activity	7/286	9.0×10^{-7}	1/6
BABA	MF	GO:0005506	iron ion binding	22/286	1.2×10^{-6}	9/13
CHI	CC	GO:0005576	extracellular region	34/245	3.5×10^{-12}	33/1
CHI	MF	GO:0004857	enzyme inhibitor activity	21/357	6.8×10^{-10}	20/1
CHI	BP	GO:0043086	negative regulation of catalytic activity	19/312	9.4×10^{-9}	18/1
CHI	BP	GO:0044092	negative regulation of molecular function	19/312	1.2×10^{-8}	18/1
CHI	MF	GO:0004869	cysteine-type endopeptidase inhibitor activity	8/357	4.4×10^{-8}	8/0
B01	MF	GO:0045735	nutrient reservoir activity	18/455	8.5×10^{-15}	18/0
B01	CC	GO:0012511	monolayer-surrounded lipid storage body	8/299	1.6×10^{-11}	8/0
B01	CC	GO:0005576	extracellular region	36/299	5.0×10^{-11}	23/13
B01	BP	GO:0019915	lipid storage	8/375	1.1×10^{-10}	8/0
B01	CC	GO:0005811	lipid droplet	8/299	1.0×10^{-7}	8/0

BP, biological process; CC, cellular component; MF, molecular function. Gene ratio = DEGs annotated to the term/all annotated DEGs. Enrichment computed from nominal DEG lists ($p \leq 0.05$, $|\log2FC| \geq 1$). SA results are shown for completeness but were not interpreted.

Table S2. Significantly enriched KEGG pathways for each treatment.

Treatment	KEGG ID	Pathway	Gene ratio	p-value	Up/Down
SA	cann04016	MAPK signaling pathway—plant	15/112	8.5×10^{-6}	10/5
SA	cann00909	Sesquiterpenoid and triterpenoid biosynthesis	6/112	1.1×10^{-4}	3/3
SA	cann04141	Protein processing in endoplasmic reticulum	14/112	3.5×10^{-4}	14/0
SA	cann00908	Zeatin biosynthesis	5/112	1.3×10^{-3}	3/2
SA	cann00520	Amino sugar and nucleotide sugar metabolism	9/112	1.7×10^{-3}	7/2
SA	cann00940	Phenylpropanoid biosynthesis	8/112	2.8×10^{-3}	2/6
SA	cann00592	alpha-Linolenic acid metabolism	5/112	3.9×10^{-3}	1/4
ASM	cann04626	Plant–pathogen interaction	27/146	7.4×10^{-10}	26/1
ASM	cann04016	MAPK signaling pathway—plant	22/146	1.4×10^{-8}	21/1
ASM	cann00480	Glutathione metabolism	13/146	8.1×10^{-6}	13/0
ASM	cann00908	Zeatin biosynthesis	7/146	7.7×10^{-5}	6/1
ASM	cann00073	Cutin, suberine and wax biosynthesis	6/146	2.4×10^{-4}	5/1
ASM	cann01040	Biosynthesis of unsaturated fatty acids	5/146	1.3×10^{-3}	5/0
BABA	cann04981	Folate transport and metabolism	6/91	3.9×10^{-5}	1/5
BABA	cann00410	beta-Alanine metabolism	5/91	1.4×10^{-3}	0/5
BABA	cann01040	Biosynthesis of unsaturated fatty acids	4/91	1.4×10^{-3}	1/3
BABA	cann00073	Cutin, suberine and wax biosynthesis	4/91	1.6×10^{-3}	3/1
BABA	cann04075	Plant hormone signal transduction	13/91	2.2×10^{-3}	2/11
BABA	cann00909	Sesquiterpenoid and triterpenoid biosynthesis	4/91	3.2×10^{-3}	3/1

BABA	cann04016	MAPK signaling pathway—plant	9/91	4.4×10^{-3}	0/9
CHI	cann00073	Cutin, suberine and wax biosynthesis	6/119	6.2×10^{-5}	6/0
CHI	cann00904	Diterpenoid biosynthesis	4/119	5.4×10^{-4}	4/0
CHI	cann04626	Plant–pathogen interaction	14/119	1.2×10^{-3}	13/1
B01	cann00073	Cutin, suberine and wax biosynthesis	8/144	2.2×10^{-6}	7/1
B01	cann04626	Plant–pathogen interaction	20/144	8.6×10^{-6}	20/0
B01	cann01212	Fatty acid metabolism	10/144	5.6×10^{-5}	10/0
B01	cann01040	Biosynthesis of unsaturated fatty acids	6/144	1.0×10^{-4}	6/0
B01	cann00940	Phenylpropanoid biosynthesis	10/144	1.3×10^{-3}	9/1
B01	cann00592	alpha-Linolenic acid metabolism	6/144	2.0×10^{-3}	4/2
B01	cann00908	Zeatin biosynthesis	5/144	4.0×10^{-3}	3/2

Gene ratio = DEGs in the pathway/all DEGs with KEGG annotation (112, 146, 91, 119 and 144 for SA, ASM, BABA, CHI and B01, respectively). Enrichment computed from nominal DEG lists ($p \leq 0.05$, $|\log2FC| \geq 1$). SA results are shown for completeness but were not interpreted.
